# Correction to: ‘Evolutionary convergence and trophic diversity in hot vent and cold seep shrimps showcase a continuum of symbiosis’ (2026), by Methou *et al.*

**DOI:** 10.1098/rspb.2026.2069

**Published:** 2026-10-07

**Authors:** Pierre Methou, Margaux Mathieu-Resuge, Loïc N Michel, Valérie Cueff-Gauchard, Hiromi Kayama Watanabe, Emily J. Cowell, Jonathan T. Copley, Roxanne A. Beinart, Magali Zbinden, Florence Pradillon, Marie-Anne Cambon, Chong Chen

**Affiliations:** ^1^ Univ Brest, Ifremer, BEEP, Plouzané, F-29280, France; ^2^ Animal Systematics and Diversity, Freshwater, and Oceanic Sciences Unit of reSearch (FOCUS), Université de Liège, Liège 4000, Belgium; ^3^ X-STAR, Japan Agency for Marine-Earth Science and Technology (JAMSTEC), Yokosuka, Kanagawa, 237-0061, Japan; ^4^ Department of Biology, Temple University, Philadelphia, PA 19122-0843, USA; ^5^ Ocean and Earth Science, University of Southampton, Southampton SO17 1BJ, UK; ^6^ Graduate School of Oceanography, University of Rhode Island, Narragansett, RI 02882, USA; ^7^ Laboratoire de Biologie des Organismes et Ecosystèmes Aquatiques (BOREA), MNHN, CNRS-8067, IRD-207, Sorbonne Universite, UCN, UA, Paris 75006, France

*Proc. R. Soc. B* **293**, 20252883. (Published online 11 February 2026). (https://doi.org/10.1098/rspb.2025.2883)

## 1. Correction details:

Regarding the geographical distribution of alvinocaridid species on the map of figure 1A, *Alvinocaris costaricensis* was mistakenly reported from Tempus Fugit and Congo Fan locations. We correct this by properly reporting geographic distribution of *Alvinocaris lusca* at Tempus Fugit vent and *Alvinocaris muricola* at the Congo fan seep. A new figure 1 is provided.

**Figure 1.**
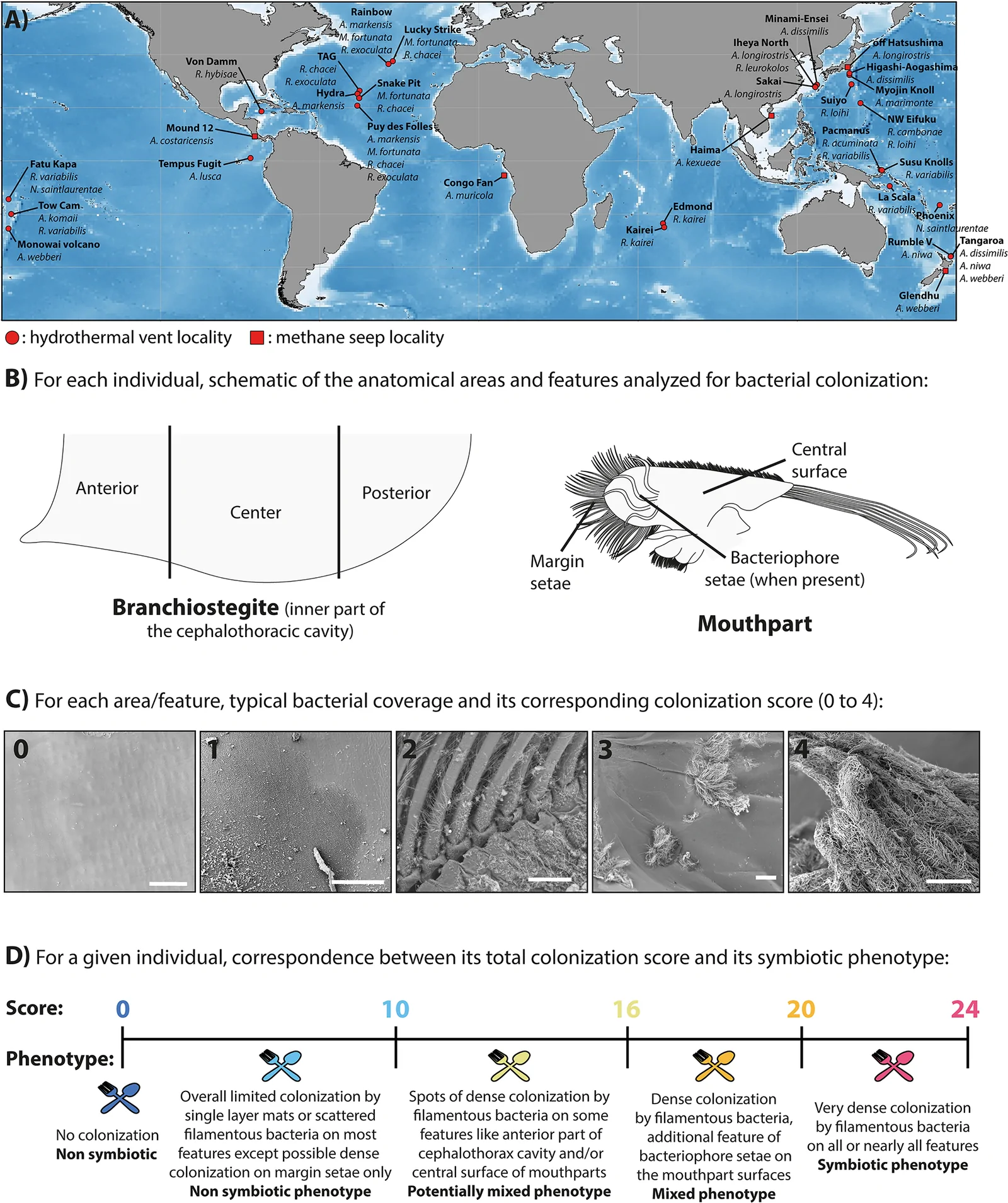
Geographic context and methodology of this study. (A) Sampling localities of alvinocaridid shrimps used in this study. This figure illustrates only the distribution of the shrimp samples used in this study and not the currently known global distribution of all alvinocarid species. (B) Schematic summarizing the method used to define bacterial colonisation for each individual with three distinct areas of the branchiostegite (anterior, center, posterior) and three features of the mouthparts (central surface, margin setae and bacteriophore setae). (C) For each area/feature, a score from 0 to 4 was attributed according to the following criteria (length of scale bars in parentheses), 0: no visible bacterial colonization (50 µm); 1: a single layer of rod-shaped bacterial mats (50 µm); 2: colonization by rod-shaped bacteria and sparsely distributed filamentous bacteria (50 µm); 3: localized spots colonized by filamentous bacteria (250 µm); 4: extremely dense colonization by filamentous bacteria, the surface of shrimp cuticle barely visible (200 µm). (D) For a given individual, a total score from 0 to 24 was defined by adding the score of each area/feature. A correspondence between the colonizing score and the supposed symbiotic phenotype is given.

